# Patient-Reported Outcomes Assessing the Impact of Palliative Radiotherapy on Quality of Life and Symptom Burden in Head and Neck Cancer Patients: A Systematic Review

**DOI:** 10.3389/fonc.2021.683042

**Published:** 2021-06-04

**Authors:** Alexander Fabian, Justus Domschikowski, Markus Hoffmann, Oliver Weiner, Claudia Schmalz, Jürgen Dunst, David Krug

**Affiliations:** ^1^ Department of Radiation Oncology, University Hospital Schleswig-Holstein, Kiel, Germany; ^2^ Department of Otorhinolaryngology, Head and Neck Surgery, University Hospital Schleswig-Holstein, Kiel, Germany; ^3^ University Library Kiel, Christian-Albrechts-University Kiel, Kiel, Germany

**Keywords:** head and neck neoplasms, squamous cell carcinoma of head and neck, palliative care, radiotherapy, patient reported outcome measures, symptom assessment, quality of life, systematic review

## Abstract

Incurable head and neck cancer has a poor prognosis and impairs a patient’s health-related quality of life. Palliative radiotherapy may improve or stabilize health-related quality of life and symptoms, best measured by patient-reported outcomes. There is no systematic analysis if palliative radiotherapy for head and neck cancer improves or stabilizes health-related quality of life or symptoms as validly measured by patient-reported outcomes. Therefore, the primary objective of this systematic review (PROSPERO-ID: CRD42020166434) was to assess the effect of palliative radiotherapy for head and neck cancer on patient-reported outcomes. The secondary objective was to assess the rate and quality of use of patient-reported outcomes in relevant studies claiming a “palliative effect” of radiotherapy. The databases MEDLINE/PubMed, EMBASE, Cochrane CENTRAL, “ClinicalTrials.gov” were searched. Concerning the primary objective, four studies were eligible to assess the effectiveness of palliative radiotherapy as measured by patient-reported outcomes. A narrative synthesis suggests a favorable impact of palliative radiotherapy on health-related quality of life and symptom burden. The risk of bias, however, is considerable and the overall quality of evidence low. Concerning the secondary objective, over 90% of studies claiming a “palliative effect” of palliative radiotherapy did either not use patient-reported outcomes or did so by limited quality. In conclusion, implementation of patient-reported outcomes in studies assessing palliative radiotherapy for head and neck cancer should be fostered. Palliative radiotherapy remains an option for head and neck cancer patients, although more studies focusing on patient-reported outcomes are needed.

**Systematic Review Registration:**

https://www.crd.york.ac.uk/prospero/, identifier CRD42020166434

## Introduction

Patients suffering from incurable head and neck cancer face a poor prognosis (1). A recent prospective clinical cohort study reported a 1-year overall survival of only 32% for head and neck cancer patients treated in “non-curative” intent (2). Even in the era of immune checkpoint-inhibitors the median overall survival is still roughly one year, for example in recurrent or metastatic cases (3, 4). In addition, severe symptoms may have a marked negative impact on a patient’s health-related quality of life (5). Palliative radiotherapy can be an option to improve or stabilize health-related quality of life and symptoms. Although palliative radiotherapy is routinely used for cancer patients in general (6), there may be a more restrictive use in head and neck cancer patients. A Canadian study suggests that compared to the average palliative cancer patient, head and neck cancer patients are more frequently referred to radiation oncology consultations, although these consultations result less frequently in actual palliative radiation (7). While the high referral rates underline the need for symptom control, low actual treatments by palliative radiotherapy indicate some degree of skepticism or other obstacles for palliative radiotherapy in head and neck cancer patients. In fact, the effectiveness of palliative radiotherapy for head and neck cancer has been questioned (8).

The main goal of any palliative treatment should be an improvement and/or stabilization of health-related quality of life and/or symptoms (9). Patient-reported outcomes are essential to measure health-related quality of life and relevant symptoms (10). Specific patient-reported outcome measures have been developed also for head and neck cancer patients, for example in the form of multi-item questionnaires (11). In order to contribute robust evidence in clinical studies, these instruments should ideally be applied in a validated, longitudinal, and prospective setting. Yet recent reviews of palliative radiotherapy for head and neck cancer synthesized any concept of palliative response; including tumor response rates or unvalidated assessments of symptom control in retrospective studies (12, 13). Hence, to date there is little guidance, if palliative radiotherapy meets the main goal of improving or stabilizing health-related quality of life or symptoms in head and neck cancer patients as validly measured by patient-reported outcomes.

Therefore, we conducted a systematic review of patient-reported outcomes of head and neck cancer patients treated with palliative radiotherapy. The primary objective was to assess the impact of palliative radiotherapy on health-related quality of life and symptoms based on patient-reported outcomes in studies using a sound methodology. The secondary objective was to evaluate the rate and quality of use of patient-reported outcomes in studies claiming a “palliative effect”.

## Materials and Methods

This is a systematic review with a narrative synthesis (14, 15). A protocol was developed on the basis of the Cochrane methodology and PRISMA statement with support of a statistician (SS) (16, 17). The protocol was registered on PROSPERO (ID: CRD42020166434) (**Supplementary Data Sheet 1**). Amendments were documented and explained on PROSPERO as far as possible at the time of publication (**Supplementary Data Sheet 2**).

### Objectives and Eligibility

The primary objective was to assess the effect of palliative radiotherapy for head and neck cancer on patient-reported outcomes before and after treatment in eligible studies. Inclusion criteria were: head and neck squamous cell carcinoma, use of palliative radiotherapy (as defined by the respective study author), prospective trial design or case series, and English language. Exclusion criteria were: radical radiotherapy, cutaneous primary, case report, no use of patient-reported outcomes, and poor quality of patient-reported outcome measurement. Poor quality of patient-reported outcome measurement was defined on the basis of a prior publication as presence of at least one of the following (18): no evidence of validity or responsiveness, not self-reported, no longitudinal assessment, no compliance data available, and time point of assessment not indicated.

The secondary objective was to assess the rate and quality of use of patient-reported outcomes in relevant studies claiming a “palliative effect” over time. The secondary objective was a post-hoc analysis decided at abstract screening stage without impact on the initial search strategy. All clinical studies of palliative radiotherapy for head and neck squamous cell carcinoma reporting a “palliative effect” were included to full text screening. The semantic term of “palliative effect” was assessed concisely. Terms like “effective palliation”, “significant palliative effect”, or “meaningful palliation” were counted; studies solely stating terms like “improvement of symptoms” were not counted.

### Search Strategy

The PICO-elements included head and neck cancer patients in a palliative setting (population), palliative radiotherapy (intervention), and health-related quality of life or common symptoms (outcome). An extensive search strategy was realized by a librarian (OW) in February 2020 and updated in November 2020 without time restriction (**Supplementary Table 1**). The databases MEDLINE/PubMed, EMBASE, Cochrane Center Register of Controlled Trials (CENTRAL), and “ClinicalTrials.gov” were searched. In addition, cross references of eligible studies were screened for relevant further studies. The online tool Covidence was used for record management. Title and abstract screening as well as full text screening for eligibility were performed independently by two authors (AF, JDo). In case of disagreement, mutual consent was found by discussion.

### Data Extraction and Data Synthesis

Concerning the primary objective, data extraction of eligible studies was done independently by two authors (AF, JDo) via predefined data extraction forms. The conduct of a meta-analysis was deemed infeasible due to the paucity of available data and due to mostly categorical outcome reporting without clear sample sizes. Instead, a narrative synthesis was performed following the framework proposed by Popay and colleagues (15). This framework for narrative syntheses consists of four iterative stages. The first stage is to develop a theory of how, why, and for whom an intervention may work. As radiotherapy is a standard treatment for many cancer patients, we limited this stage to the description provided in the second paragraph of the introduction. The second stage, conducting a preliminary synthesis of findings of included studies, was performed via tabulation in the findings section (**Table 3**) (19). The table is ordered by the first author’s name of the study. The groups and domains of patient-reported outcomes displayed in **Table 3** represent all validly reported patient-reported outcomes of the eligible studies. One study reported two additional domains that are not displayed in **Table 3** due to a lack of comparability with the other included studies for these domains (20). Results of patient-reported outcomes are categorically displayed in **Table 3** as reported by the respective study. Vote counting based on the direction of effects in patient-reported outcomes was performed in order to avoid subjective synthesis rules. The reported data of included studies did not allow for other synthesis methods or standardized metrics (19). The binary distinction between harm and benefit in the vote counting process was defined as benefit if more than 50% of the patients in an individual study reported “improved” or “unchanged” results for a respective patient-reported outcome domain. The third stage is to explore relationships within the findings. A textual exploration is provided in the findings section. The exploration was performed in collaboration by three authors (AF, JDo, DK). Due to the paucity of eligible studies and available data resulting in adequate tabular display, we did not provide a graphical overall synthesis. The fourth stage is to assess the robustness of the synthesis. For this purpose, the overall quality of evidence for patient-reported outcomes was assessed independently by two authors (AF, JDo) per GRADE criteria (21). The ROBINS-I tool was used independently by two authors (AF, JDo) to evaluate the risk of bias of included individual non-randomized studies (22). A formal risk of bias assessment across studies was not performed, as this mostly relies on quantitative summary data (23). Finally, Popay and colleagues suggest to critically reflect on the synthesis process and to provide conclusions and recommendations as end of the synthesis (15). Both steps are provided in the discussion section.

Concerning the secondary objective, data extraction was performed independently by two authors (AF, JDo). Clinical studies stating a “palliative effect” were counted and assessed for use of patient-reported outcomes. If patient-reported outcomes were used, their measurement was assessed for validity, compliance data, availability of baseline assessment, and indication for time of assessment.

Any disagreements in the data extraction and synthesis processes were resolved by discussion among the authors (AF, JDo, DK).

## Results

### Screening Results

**Figure 1:**
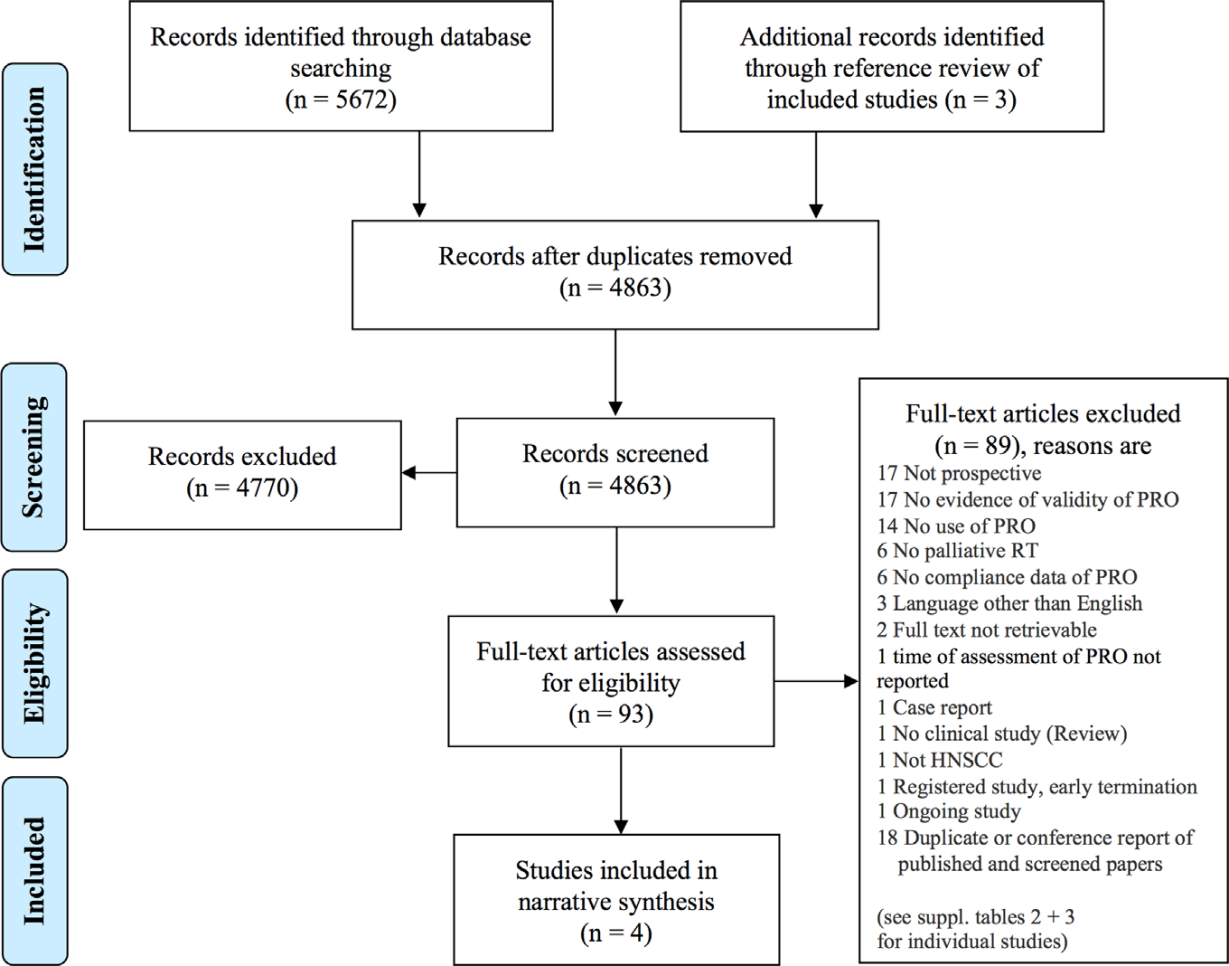
PRISMA flow diagram (17). The databases MEDLINE/Pubmed, EMBASE, Cochrane CENTRAL, and “ClinicalTrials.gov” were searched. Screening was performed independently by two authors. HNSCC, head and neck squamous cell carcinoma; PRO, patient-reported outcome; RT, radiotherapy.

The database search and assessment of cross references resulted in 4863 records to screen for title and abstract as shown by the PRISMA flow diagram (**Figure 1**) (17). Ninety-three records were included to full text screening. Four studies were eligible for the primary objective and included in the narrative synthesis (20, 24–26). The most prevalent reasons for exclusion were unvalidated patient-reported outcomes, no use of patient-reported outcomes, and no prospective study design (see **Supplementary Tables 2** and **3**).

### Use of Patient-Reported Outcomes in Studies Reporting a “Palliative Effect”

**Figure 2:**
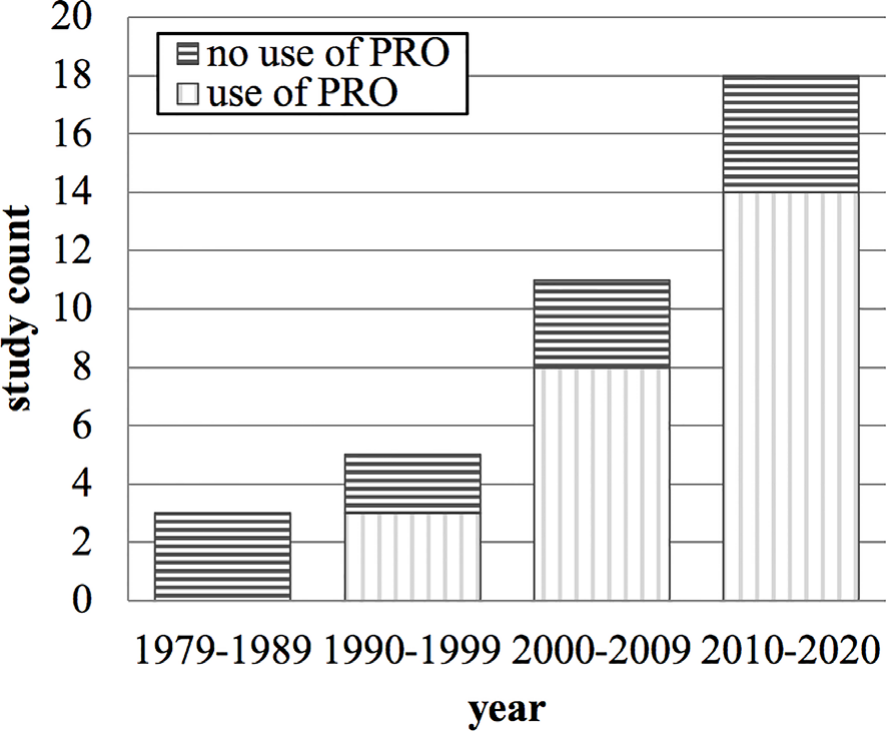
Count of clinical studies stating a “palliative effect” of palliative radiotherapy for head and neck cancer and their use of patient-reported outcomes over time in years. PRO, patient-reported outcome.

Concerning the secondary objective, 37 clinical studies reported a “palliative effect” of palliative radiotherapy for head and neck cancer patients (**Table 1**). Of these studies, 12 (32%) did not use patient-reported outcomes. Twenty-two studies (60%) applied patient-reported outcome measurement with limited quality; “no evidence of validity” and “compliance not reported” being the most common reason. The remaining three studies (8%) are three out of the four eligible studies mentioned above (20, 24, 26). From 1979 to 2020, the number of clinical studies reporting a “palliative effect” of palliative radiotherapy for head and neck cancer patients increased per decade (**Figure 2** **)**. From 2010 to 2020, 22% of these studies did still not report patient-reported outcomes.

**Table 1:** Use of patient-reported outcomes and reasons for limited quality of their assessment in studies reporting a “palliative effect”.

Characteristic	Number of studies	References
Total number of studies stating a “palliative effect”	37 (100%)	(20, 24, 26–60)
No use of PRO	12 (32%)	(27–31, 36–38, 43, 44, 47, 51)
		
Use of PRO with limited quality	22 (60%)	(32–35, 39–42, 45, 46, 48–50, 52–60)
no evidence of validity	17	(32–35, 41, 42, 46, 48–50, 52, 54, 55, 57–60)
compliance not reported	17	(34, 35, 42, 45, 46, 48–50, 52, 54–60)
time of assessment not reported	14	(32, 34, 35, 45, 46, 48, 50, 52, 53, 55–58, 60)
no baseline assessment before RT	7	(32, 34, 40, 42, 46, 48, 60)

Clinical studies, including conference abstracts, were counted that report a “palliative effect” of a palliative radiotherapy regimen for head and neck cancer patients. Per protocol, these studies were implicitly selected for full text screening. The total number comprises three studies included in the narrative synthesis of the primary objective.

PRO, patient-reported outcome; RT, radiotherapy.

### Characteristics and Findings of Eligible Studies for the Primary Objective

#### Preliminary Synthesis

The characteristics of all four eligible studies for the primary objective are shown in **Table 2**. Three were prospective, non-randomized phase II studies (20, 24, 26). One study was a prospective radiation dose-escalation study (25). Quality of life was the primary endpoint in only one study (26). All studies were conducted in high-income countries. No patient had prior radiotherapy. There was no concurrent systemic therapy per protocol in any study. All trials studied hypofractionated external beam radiotherapy. One study assessed a cyclical regimen known as “Quad Shot” with four fractions in two consecutive days repeated every four weeks for up to three cycles (24). Another study assessed a similar protocol at higher radiation doses (25). The remaining two studies employed once daily consecutive or intermittent fractions (20, 26). Toxicity of grade three or higher was absent in two studies (24, 25), moderate (e.g. mucositis 7%) in one study (26), and more pronounced (e.g. mucositis 26%) in the remaining study (20). The progression-free survival ranged from 3.1 to 3.9 and the overall survival from 5.7 to 6.5 months, respectively.

**Table 2:** Characteristics of eligible studies.

Study	Corry et al., 2005 (24)	Ferro et al., 2020 (25)		Fortin et al., 2016 (26)		Porceddu et al., 2007 (20)	
**Design**	prosp., phase II, single-arm, single-center	prosp., dose escalation, single-center		prosp., phase II, single-arm, multi-center (2)		prosp., phase II, single-arm, multi-center (4)	
**Patient characteristics**							
Incl. patients	30	17		32		37	
Country	Australia	Italy		Canada		Australia	
Median age	72	85		74		68	
Sex (m:f)	73:27	59:41		84:16		81:19	
Performance status (ECOG)	0: 7%/1: 27%/ 2: 43%/3: 23%	0-1: 29%/2: 24%/3: 47%		1: 61%/2: 39%		0: 19%/1: 51%/ 2: 24%/3: 5%	
Main tumor sites	Oral cavity: 43% Oropharynx: 27% Hypopharynx: 20%	Oropharynx: 24% Larynx: 24% Oral cavity: 18%		Oropharynx: 28% Larynx: 13% Oral cavity: 13%		Oropharynx: 32% Oral cavity: 27% Hypopharynx: 16% CUP: 16%	
Main tumor stages	UICC IV: 97%	UICC IV: 53%		T4, N3 or M1: 66%		UICC IV: 65%	
**Intervention**							
RT regimen	14 Gy in 4 fx in 2 days q4w for max. 3 cycles	20 Gy in 4 fx in 2 days q4w for max. 2 cycles		25 Gy in 5 consecutive daily fx		30 (or 36) Gy in 5 (or 6) fx twice weekly	
RT technique	2D-conventional	3D-conf./IMRT		IMRT		3D-conformal	
Compliance – RT as planned p.p.	1 cycle: 20% 2 cycles: 27% 3 cycles: 53%	100%		88%		84%	
Median f/u	not reported	4 months		12.2 months		21 months	
**Outcomes – except PRO**							
Toxicity ≥°III	Worst during entire follow-up (CTC v2): °III/IV: none	Worst during entire follow-up (RTOG): °III/IV: none		Worst during entire follow-up (CTC v4): °III Mucosal: 7% °III Other: 7% °IV: none		Acute toxicity (CTC v2): °III Skin: 11% °III Mucositis: 26% °III Dysphagia: 11% °III Salivary: 6% °IV Dysphagia: 6%	
Median PFS	3.1 months	not reported		3.2 months		3.9 months	
Median OS	5.7 months	not reported		6.5 months		6.1 months	
CoI – “notable concern”	No	No		No		No	

CTC, Common Toxicity Criteria; CoI, conflict of interest; CUP, cancer of unknown primary; ECOG, Eastern Cooperative Oncology Group; fx, fraction; f/u, follow-up; IMRT, intensity-modulated radiotherapy; OS, overall survival; PFS, progression-free survival; p.p., per protocol; w, week; RT, radiotherapy.

**Table 3** displays the summary of findings for patient-reported outcomes of all four eligible studies. The studies employed different patient-reported outcome measures. One study used linear analogue scales for three domains and the visual analogue scale for the domain “pain” (25). The other studies used multi-item questionnaires (20, 24, 26). Of note, one study modified the questionnaire extensively resulting in only one validly measured outcome domain (“global quality of life”) (24). All studies scheduled multiple assessments after the completion of radiotherapy for comparison to baseline. One study reported only outcomes for the first follow-up (25). Two studies reported only the best results at any follow-up (20, 24). The remaining study provided multiple outcomes for a follow-up period of six months (26). In order to allow for better comparison between studies, we have chosen to display the best results after the completion of radiotherapy for the latter study. Results of comparable patient-reported outcome domains are juxtaposed in **Table 3**, as the instruments used share comparable constructs for (head and neck) cancer patients. The specific domain definition may, however, differ for some outcomes per instrument used. For example, the domain “physical functioning” refers to “ability to perform daily activities” (25), “physical functioning” (26), or “ability to work” (20). Per vote counting, as defined in the methods section, palliative radiotherapy resulted in a benefit for all patient-reported outcome domains across the studies (**Table 3**). This is also the case for all time points in the study that reported multiple assessments in a six month follow-up period (data not displayed in this review) (26).

**Table 3:** Summary of findings table for patient-reported outcomes of eligible studies.

Study	Corry et al., 2005 (24)	Ferro et al., 2020 (25)	Fortin et al., 2016 (26)	Porceddu et al., 2007 (20)
**PRO measures**				
Instrument	EORTC-QLC-C30 (modified)	VAS (pain), CLAS	EORTC-QLQ-C15-PAL and H&N35	FACT – H&N
Characteristic	Validated core questionnaire for QoL; modified by study authors; only global QoL validly assessable	Validated scales for symptom or QoL domains; VAS range 0-10 (higher score = higher burden)	Validated questionnaires for QoL; core module for palliative cancer patients + module for HNC	Validated questionnaire for QoL; head and neck cancer specific
Missing data for analysis	17% (patients not filling out at least 1 pre- and post-RT questionnaire)	0% (for baseline and first follow-up)	14% (questionnaires not completed)	34% (patients not filling out at least 2 post-RT questionnaires)
Time of assessments	Baseline, d2 of RT-cycles, w2 after RT-cycles, q3m m3-9 after final RT	Baseline, w3 after RT-cycles, q2m after final RT	Baseline, q1m for m1-6, q2m m8-12, q3m m15-24	Baseline, q1m for m1-3, q3m m6-until close out date or death
**Results of comparable PRO domains**				
Comparison	Best results in post-RT period *vs.* baseline	3 weeks post RT *vs.* baseline	Best results in post-RT period *vs.* baseline	Best results in post-RT period *vs.* baseline
Global quality of life	44% better 28% unchanged 28% worse	29% better 35% unchanged 36% worse	76% better or unchanged 24% worse	62% better 14% unchanged 24% worse
Pain	n/a	General: VAS pre-RT: 3.9 VAS post-RT: 2.7 (mean, p=.037)	Head and neck: 83% better or unchanged 17% worse	Head and neck: 76% better 14% unchanged 10% worse
Swallowing	n/a	n/a	75% better or unchanged 25% worse	43% better 38% unchanged 19% worse
Fatigue	n/a	41% better 29% unchanged 30% worse	87% better or unchanged 13% worse	29% better 24% unchanged 48% worse
Physical functioning	n/a	24% better 35% unchanged 41% worse	76% better or unchanged 24% worse	76% better 0% unchanged 34% worse
**Quality of evidence for PRO**	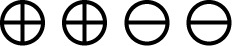 **LOW^1^** per GRADE-Criteria, due to study design and risk of bias			

^1^The level of evidence was not downgraded further as all studies applied precise eligibility criteria. CLAS, cancer linear analogue scale; d, day; HNC, head and neck cancer; m, month; n/a, not applicable; PRO, patient-reported outcome; RT, radiotherapy; VAS, visual analogue self-assessment scale; w, week.

#### Relationships Within the Findings

**Tables 2** and **3** offer insights into relationships within the findings with regard to patient-reported outcomes. Yet if differences in patient-reported outcomes reflect relations to study characteristics, should only cautiously be explored on a hypothesis-generating basis. This is due to small sample sizes, reporting of categorical rather than absolute changes, and heterogeneity in the reported comparison to baseline of patient-reported outcomes. Nevertheless, three relating aspects may be identified.

First, differences in effect sizes of specific outcome domains could in part relate to different patient-reported outcome measures used in the studies. The domain fatigue is, for example, a composite score in the EORTC-QLQ-C15-PAL (26), whereas it refers to a single question on “lack of energy” in the FACT-H&N questionnaire (20). Second, the results of patient-reported outcomes tend to be inferior in one study (25) compared to the other studies (20, 24, 26). This might relate to the patient’s significantly higher age as well as to the comparison to baseline (25); only the first follow-up was reported instead of the best result within multiple assessments. Third, a relevant fraction of patients in all studies reported worsened health-related quality of life scores despite limited treatment-related toxicity. This aspect may be related to advanced disease stages, the fact that all radiotherapy protocols treated only the symptomatic gross disease in order to limit toxicity, and short progression-free survival. Competing tumor growth may therefore be related to worsened health-related quality of life scores in some patients.

#### Robustness of the Synthesis

**Figure 3:**
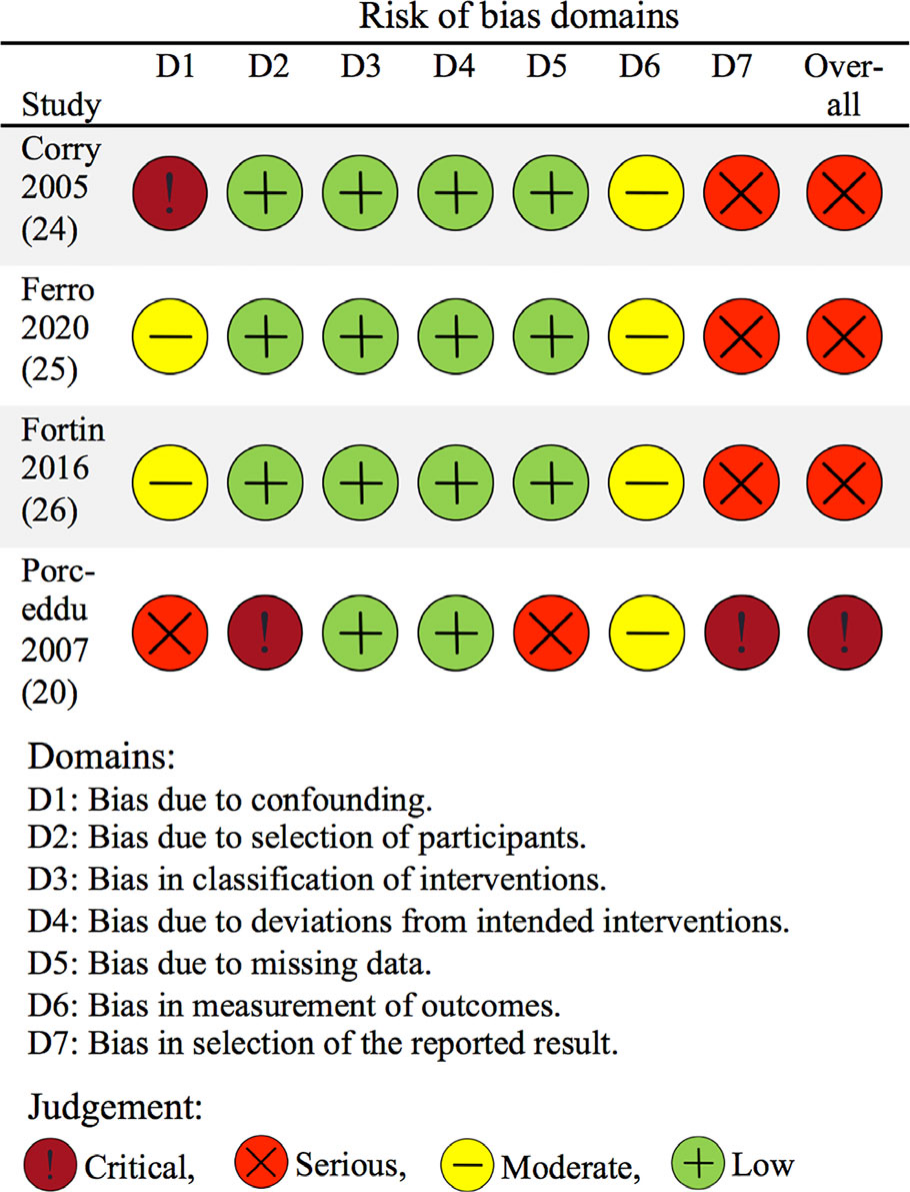
Risk of bias of included studies concerning patient-reported outcomes. The risk of bias of included studies was assessed using the ROBINS-I tool for non-randomized studies (22). The figure is based on the “robvis” tool (61).

The overall quality of evidence for a positive impact of palliative radiotherapy for head and neck cancer on health-related quality of life or symptoms as measured by patient-reported outcomes was “low” per GRADE-criteria (**Table 3**). The risk of bias assessment of included, individual studies showed “serious” overall risk of bias in three studies (24–26) and “critical” overall risk of bias in one study (20) per ROBINS-I tool (22, 61) (**Figure 3**).

#### Registered Studies

Relevant studies that are registered on “ClinicalTrials.gov”, but were not reported in November 2020, are displayed in **Table 4**. One study was terminated early due to poor recruitment. One ongoing prospective study uses the patient-reported outcome measure “EORTC-QLQ-C15-PAL” and awaits primary completion in November 2022.

**Table 4:** Studies registered on “CinicalTrials.gov” and not yet reported in November 2020.

Study identifier	Study Start Date	Planned enrollment	Actual enrollment	Comparators	PRO used	Status
NCT03637335	08/2015	NR	26	10x3 Gy + carboplatin *vs.* 10x3 Gy + placebo	EORTC-QLQ- C30, VAS	Early termination due to poor recruitment
NCT03804073	11/2017	82	NR	10x3 Gy *vs.* 20 Gy in 4 fx in 2 days	EORTC-QLQ- C15-PAL, Likert scales	Recruiting, primary completion in 11/2022

List of studies evaluating palliative radiotherapy for squamous cell head and neck carcinoma by patient-reported outcomes. All studies are prospective, randomized, multicenter and based in Europe.

fx, fraction; NR, not reported; PRO, patient-reported outcome; VAS, visual analog scale.

## Discussion

This systematic review of patient-reported outcomes in patients treated with palliative radiotherapy for head and neck cancer shows that over 30% of studies claiming a “palliative effect” did not use patient-reported outcomes. Sixty percent of the studies applied patient-reported outcomes with limited quality, for example owing to unvalidated instruments or unreported compliance data. Four studies were eligible to analyze the primary objective: the effectiveness of palliative radiotherapy as validly measured by patient-reported outcomes. While a planned meta-analysis or calculation of summary effect sizes was not feasible, all studies suggested a positive impact of palliative radiotherapy on global health-related quality of life. In addition, relevant symptoms seemed to be alleviated or stabilized. The risk of bias, however, was high and the overall quality of the evidence concerning patient-reported outcomes remains low per GRADE-criteria.

The low rate of patient-reported outcomes and standard of their use in studies of palliative radiotherapy for head and neck cancer is in part also reflected in other entities. In fact, a systematic review of palliative radiotherapy for rectal cancer showed that none of 27 eligible studies assessed patient-reported outcomes (62). Furthermore, a systematic review of palliative radiotherapy for prostate cancer could neither identify any study applying patient-reported outcomes (63). Another systematic review evaluated patient-reported outcomes in palliative radiotherapy for esophageal cancer (18), a patient population sharing risk factors with head and neck cancer patients. Only six studies conducted patient-reported outcome assessment of sufficient standard as defined by the review authors. Next to these overall relatively low rates of patient-reported outcome evaluation, a recent systematic review on patient-reported outcome measures in palliative radiotherapy studies across entities confirmed quality issues, for instance in patient-reported outcome reporting (64). Hence, our finding is reflective of the current literature and might be rooted in a long-recognized difficulty in defining the right outcome measure for palliation (65). Patient-reported outcomes, however, are generally accepted to measure health-related quality of life and symptom burden, also in a palliative setting (66).

Even though patient-reported outcome measures are the cornerstone to assess health-related quality of life and symptom burden, their implementation is challenging. A major concern is missing data. In fact, it has long been recognized that missing patient-reported outcome data represent not only random effects but also important information (67). For example, missing data could result from a missed patient visit that was cancelled due to a deterioration of the patient’s health. In order to allow for a meaningful interpretation of patient-reported outcomes, the rate of missing data should be as low as possible. The rate of missing patient-reported outcome data for analysis was 34% in one of the eligible studies for our primary objective (**Table 3**) (20). This might raise the question if patient-reported outcome measures are an appropriate tool to assess health-related quality of life and symptom burden in patients with incurable head and neck cancer. In fact, missing data in trials of palliative interventions is an issue for most endpoints. A systematic review and meta-analysis has reported a rate of 23% of missing data for all primary endpoints in randomized trials of palliative interventions (68). Health-related quality of life was even associated with significantly higher rates of missing data in the meta-analysis. In this light, one of the eligible studies for the primary endpoint of our review reported only 14% of missing patient-reported outcome data for all scheduled questionnaire assessments (26). This demonstrates that the rate of missing patient-reported outcome data can be lower than average in head and neck cancer patients undergoing palliative radiotherapy. In our view, the use of patient-reported outcome measures should therefore not be discouraged in this setting.

Despite limited evidence in terms of validly measured patient-reported outcomes regarding the effectiveness of palliative radiotherapy for head and neck cancer, recent data demonstrate the frequent use of radiotherapy in this context. A British prospective cohort study of head and neck cancer patients treated at 76 UK centers reported that over 50% of those treated in “non-curative” intent received radiotherapy (2). This goes in line with guidelines suggesting palliative local therapy such as radiotherapy for head and neck cancer patients when indicated (69, 70). Nevertheless, the burden of radiotherapy-related toxicity should be carefully considered. Most included studies for our primary objective reported remarkably low toxicity rates (24–26), which may have less negative impact on health-related quality of life compared to more intensive radiotherapy regimens. In light of limited prognosis, toxicity is crucial, as patients with head and neck cancer and palliative patients in general show the highest rates of radiotherapy-associated hospital admissions (71). Keeping in mind that health-related quality of life should be a main outcome, a review of palliative radiotherapy for head and neck cancer also judges patient-reported outcomes essential to optimize decisions on therapy (13). The eligibility criteria for the primary objective of our study were designed in order to ensure an appropriate quality of studies reporting patient-reported outcomes, the latter being a crucial aspect as stated by the Cochrane foundation (72). All four eligible studies reported a positive impact of palliative radiotherapy on health-related quality of life and relevant symptoms. Another systematic review on palliative radiotherapy for head and neck cancer used less stringent eligibility criteria. This review included for example retrospective studies assessing local tumor control rates or unvalidated patient-reported outcome measures (12). Nevertheless, the studies presented there are as well in favor of palliative radiotherapy. The question remains, however, how to define the patient population that may benefit from palliative radiotherapy for symptomatic head and neck cancer instead of radical radiotherapy or also omission of radiotherapy. Put simply as a framework, this question relates to four key aspects: i.) comorbidity and/or performance status, ii.) tumor extension, iii.) prognosis as judged clinically, and iv.) an individual patient’s preferences. These aspects need to be merged and weighed individually in a shared decision-making process. Even in a similar clinical situation, this process may result in divergent decisions reaching from a more radical to a palliative radiotherapy approach or also omission of radiotherapy.

Our systematic review has limitations. The number of eligible studies for the primary objective was small. This precluded a meta-analysis and reduces the confidence in the evidence. The narrative synthesis conducted instead followed a methodological framework (15) but inflicted in part personnel judgement of the authors. This is for example reflected in the choice of findings to be explored regarding their potential relationships. Furthermore, two studies only reported the best results of patient-reported outcomes after completion of radiotherapy (20, 24). This approach favors the display of a positive effect of radiotherapy. One study, however, reported multiple assessments after radiotherapy that all showed a benefit of palliative radiotherapy per vote counting (26). Next, the sample sizes of all eligible studies are small. As head and neck cancer is a heterogeneous and relatively rare disease in Western countries, large sample sizes are difficult to achieve. This is also shown by one European study closed early due to poor recruitment (73), while only one ongoing study is listed on “ClinicalTrials.gov” (**Table 4**). Moreover, most head and neck cancer cases are located in low- and middle-income countries in Asia and on the Indian subcontinent (74). Palliative radiotherapy for head and neck cancer is of major importance in this setting. Yet no local study met our eligibility criteria, in part due to linguistically unvalidated patient-reported outcome measures. On the other hand, one of the strengths of our systematic review is the emphasis on health-related quality of life and symptom burden. This is essential in the palliative setting and of greater relevance than surrogate measures such as radiographic response rates. Furthermore, we applied strict eligibility criteria for studies reporting patient-reported outcomes. In contrast to earlier reviews (12, 13), this approach allows for a meaningful minimum standard in the critical appraisal of the evidence. Finally, to our knowledge, we are the first to use certain tools of high standard for risk of bias assessment [ROBINS-I (22)] and overall evidence assessment [GRADE (21)] in our study setting.

In conclusion, the overall quality of evidence concerning the effectiveness of palliative radiotherapy for head and neck cancer is low as measured by patient-reported outcomes. Nevertheless, existing evidence suggests a positive impact on health-related quality of life and symptom burden. Although further validation by studies including patient-reported outcomes is urgently needed, palliative radiotherapy should be considered in appropriate cases.

## Data Availability Statement

The original contributions presented in the study are included in part in the article/**Supplementary Material**. Further inquiries can be directed to the corresponding author.

## Author Contributions

The protocol was developed by AF, JDu, OW, and DK. The search strategy was realized and conducted by OW. Screening, data extraction, and data synthesis were performed by AF, JDo, and DK. Figures, tables, and manuscript were compiled by AF and JDo and substantially revised and adapted by JDu, MH, CS, and DK. All authors contributed to the article and approved the submitted version.

## Funding

We acknowledge financial support by Land Schleswig-Holstein within the funding program “Open Access Publikationsfonds”.

## Conflict of Interest

DK has received honoraria from Merck Sharp & Dome.

The remaining authors declare that the research was conducted in the absence of any commercial or financial relationships that could be construed as a potential conflict of interest.
