# Supplementary material for: Patient-Reported Outcomes Assessing the Impact of Palliative Radiotherapy on Quality of Life and Symptom Burden in Head and Neck Cancer Patients: A Systematic Review
Source: Front Oncol. 2021 Jun 4;11:683042. doi: 10.3389/fonc.2021.683042 (PMC8213366; doi:10.3389/fonc.2021.683042)
Supplement: Supplementary file 1 [file DataSheet_1.docx]

**Patient-reported outcomes for health-related quality of life in head and neck cancer patients treated with palliative radiotherapy:
protocol of a systematic review and planned meta-analysis**

A. Fabian^a^#, J. Domschikowski^a^, F. Pyschny^a^, J. Dunst^a^

^a^ Department of Radiation Oncology, University Hospital Schleswig-Holstein (UKSH), 24105 Kiel, Germany

# Correspondence address: Alexander Fabian MD, Department of Radiation Oncology, University Hospital Schleswig-Holstein (UKSH) Campus Kiel, Arnold-Heller-Str. 3, 24105 Kiel, Germany, Tel.: +4943150026501,
Fax: +4943150026564, alexander.fabian@uksh.de

## Registration

Registration is planned in the “PROSPERO” registry.

## Author contribution

AF wrote the protocol. AF, FP, and JD will be engaged in screening the eligible literature, data extraction and synthesis, assessing risk of bias and drafting the manuscript.

## Support

There is no external funding source or sponsor.

## Index

[**1. Introduction**](#_nw1yxnzdr0r0) **3**

[1.1. Rationale](#_tpwo5z6q7myo) 3

[1.2 Objectives](#_to8k5oj50zme) 3

[**2. Methods**](#_x1f11rz1yo2g) **4**

[2.1 Eligibility](#_4j4opnx3cagf) 4

[2.2 Information sources and search strategy](#_o2qu4r8l84cc) 4

[2.3 Study records](#_ncgnq9ej6ptq) 4

[2.4 Outcomes and prioritization](#_iytj756hq5cw) 6

[2.5 Risk of bias in individual studies](#_7rxl6hbqi1z0) 7

[2.6 Data synthesis](#_87prklbzv0ry) 7

[2.7 Meta-biases](#_804u34phy2yh) 8

[2.8 Confidence in cumulative evidence](#_uzxejyy3a997) 8

[**3. Abbreviations**](#_m4kqcvpqoor) **9**

[**4. Appendix**](#_9ie7an8kvr93) **9**

[**5. References**](#_v4zdxa94nltg) **9**

## 1. Introduction

### 1.1. Rationale

Patients undergoing palliative treatment for head and neck cancer (HNC) face a dismal mean survival of roughly 6 months to one year (1,2). Despite some improvements this hasn’t changed dramatically in the era of immunotherapy (3). Hence, improvement or stabilization of health-related quality of life (Hr-QoL) is paramount. Hr-QoL is best assessed by “patient-reported outcomes” (PROs) as the interpretation by caregivers may differ significantly (4). To investigate PROs, validated tools (e.g. questionnaires) have been developed called “patient-reported outcome measures” (PROMs).

Radiotherapy (RT) is frequently used to palliate symptoms in HNC patients. A plethora of different palliative radiation regimes has been proposed (5). However, the effect of palliative radiotherapy on Hr-QoL as measured by PROs has not yet been systematically summarised. Yet, this is arguably the most important outcome measure for HNC patients irradiated with palliative intend. A systematic review and meta-analysis of high quality Hr-QoL data in terms of PROs could ideally guide caregivers to: i.) judge if RT robustly improves or stabilizes Hr-QoL, ii.) choose the right RT regime for their patient, and iii.) stress the need of further research in Hr-QoL in case of lacking data.

### 1.2 Objectives

The context for this review are HNC patients treated with palliative RT. The overall use of PROMs as indicator for Hr-QoL will be assessed in clinical studies. A trend over time in the use of PROMs in these studies should also be investigated. The main objective is to analyse longitudinal (pre- vs. post-treatment) differences in Hr-QoL as measured by validated PROMs in dependence of palliative RT in general. First, changes in overall scores of generic or disease specific instruments (e.g. EORTC QLQ C-30, EORTC QLQ HN-35) will be analysed per instrument. Second, changes for specific typically relevant symptoms will be assessed per symptom (6). Furthermore, differences in changes of PROMs in dependence of different RT regimes will be investigated in case of sufficient comparability. Additionally, studies referring to the explicit term ”quality of life” as an outcome but without any patient-reported outcome will be quantified. The latter will concern studies meeting the inclusion and exclusion criteria except “quality of PRO measurement”. Finally, causes of low quality of PROMs in studies excluded for a potential meta-analysis will be noted.

## 2. Methods

### 2.1 Eligibility

Inclusion criteria comprise HNC, use of RT (as defined and clearly stated by the authors) in palliative setting for the HNC, assessment of quality of life, prospective randomised or non-randomised trial design or case series, and study reports in English language.

Exclusion criteria are radical radiotherapy regime, cutaneous tumor as primary, no use of PRO or poor quality of PRO measurement (no published reports of validity or responsiveness, not self-reported, no longitudinal assessment, no compliance data available, time point of assessment not indicated ), and case reports.

Studies reporting on a subset of eligible patients will be included if separate data from this subset can be retrieved from the full text publication.

### 2.2 Information sources and search strategy

Sources to screen are PubMed, EMBASE dataset and Cochrane Central Register of Controlled Trials (CENTRAL) for eligible studies reported until date of search. A draft of the search strategy is presented in Appendix 1. Duplicates will be removed. Reference lists of included studies will be screened for further eligible studies. To reduce non-reporting bias, conference abstracts will also be considered for inclusion. Additionally the trial register “ClinicalTrials.gov” will be screened and outcome data will be retrieved if applicable. Ongoing trials will be listed in a table.

### 2.3 Study records

It is planned to use “Covidence” as program to manage records and data. Search results of the different databases will be merged. First, two authors will independently screen studies based on title and abstract for potential inclusion eligibility and discuss it in case of divergent interpretation. Second, full text will be retrieved. Third, at least two authors will independently judge eligibility based on full text and again discuss in case of divergent interpretation. Exclusion based on poor outcome measurement of quality of life data will be categorised and reported. Different reports of the same study will be linked together. In case of missing essential information for conduct of a meta-analysis authors of the study will be contacted. Ongoing but not yet reported studies will be tagged and summarised in a table. Data from studies will be extracted based on full text and correspondence with authors in case of missing essential information. Two authors will independently extract data into two prespecified data collection forms linked to an author-ID. The first form will serve to categorise studies using PROs with insufficient quality of their use and sufficient quality (variables displayed in Table 1). The second form will be used for included studies to extract data on patient characteristics, study characteristics, intervention (RT), outcomes (PROM, toxicity, OS), judgement on notable conflict of interests and risk of bias (variables displayed in Table 2+3). The versions of data collection forms of both authors will be compared and discussed in case of discrepancies. The data collection forms will be pilot-tested for usability and completeness.

Table 1: List of variables for eligibility criteria and PRO assessment quality

**study identification**
first author
year published *yyyy*

**inclusion criteria**all inclusion criteria met *yes / no* **exclusion criteria**case report, radical RT, *yes / no*
cutaneous primary

**quality of PRO assessment**evidence of validity *yes / no*
evidence of responsiveness *yes / no*
self-reported *yes / no*
compliance reported *yes / no*
pre + post RT assessment *yes / no*
time of assessment reported *yes / no*

**QoL stated as outcome without use of PRO** *yes / no*

Table 2: List of variables except outcomes

**study identification**
first author
year published *yyyy*

**patient characteristics**
number of patients
country *e.g. USA*
mean age
sex (m:f)
comorbidity *e.g. CharlsonComorbidityIndex*
mean karnofsky or ECOG
 pre-treatment
radiotherapy *yes / no*
surgery *yes / no*
systemic therapy *yes / no*
 tumor
three main tumor sites *in numbers*
tumor stages per UICC *in numbers*

**radiotherapy**total dose *in Gy*
dose per fraction *in Gy*
fractions per day *number*
fractions per week *number*
timing *cyclical or continuous*
treatment duration *in days*technique *2D, 3D, IMRT etc.*
target volume limited
 to gross tumor volume *yes/no*
integrity *RT as planned p.p*.

**study design**
study type *random./prosp…*
involved centers *single, multi*
follow up *in months*
duration *in years*

**conflict of interest**
notable concern *yes / no*rationale for judgement

**risk of bias**
RoB2 result *if randomised*
Robins-I result *if non-randomised*

###

### 2.4 Outcomes and prioritization

Main outcomes will be data for PROMs (variables displayed in Table 3). PROMs will be divided in overall scores based on generic or disease specific multi-item questionnaires for HR-QoL (e.g. FACT-HN) and single-item PROMs assessing specific symptoms (e.g. Numeric Rating Scale for pain). Commonly used questionnaires that will be searched are listed in the search strategy (7). Assessment of PROMs evaluating specific symptoms are based on a published patient-reported core set of symptoms for head and neck cancer patients (6). The focus on PROM score assessment will be directed to the comparison of pre- and post-radiation values. Post-radiation values will be pooled and are defined as “first assessment after completion of RT”. However, the time of assessment will also be analysed and might influence adjustments of the “post-radiation value data pool” in case of multiple assessments and significant heterogeneity concerning timing of assessment. In addition the “first assessment after completion of RT” should be at the earliest after 4 weeks of completion so that acute toxicity could regress. In case of multiple and similar PROMs within one study (e.g. EORTC-H&N35 and FACT-HN) the best validated PROM will be chosen for interpretation and the judgement will be justified in supplementary data.

Additional outcomes will be toxicity measures and overall survival. These will help to put PROM data into perspective but have been reviewed elsewhere (5).

Table 3: List of variables for outcomes

**study identification**
first author
year published *yyyy*

**PROM multi-item questionnaires - overall
 scores**
measure *e.g. FACT-HN*
description *generic / disease
 specific, range,
 low/high better QoL?*
score pre RT *+ standard deviation*
first score post RT *+ standard deviation*
time of assessment post RT *e.g. 6 weeks*
missing data *e.g. 7 patients*

**toxicity - CTCAE**
≥ grade 3 *total in %*
most common *e.g. mucositis °III 32%*
therapy associated *in numbers*
 hospitalisation

therapy associated *in patient numbers*
 tube feeding

**PROM - specific symptom**domain e.g. *pain*measure *eg. pain NRS*
description *range,
 low/high better QoL?*
score pre RT *+ standard deviation*
first score post RT *+ standard deviation*
time of assessment post RT *e.g. 6 weeks*
missing data *e.g. 7 patients*

**survival**
mean *in months*
 overall survival

###

### 2.5 Risk of bias in individual studies

Risk of bias of reported main outcomes will be assessed according to the “RoB 2.0” tool for randomised trials and “ROBINS-I” tool for non-randomised trials if possible (8,9). The risk of bias for each study will be displayed in the relevant tables.

### 2.6 Data synthesis

Characteristics of included studies (PICO elements) will be summarised in a table and critically reported with respect to the degree of evidence. Suitable and similar studies and outcomes (PROM questionnaires for overall scores for Hr-QoL and PROM for specific symptoms) will be grouped for synthesis. Ideally, studies using the same PROM will be grouped, pooled, and results will be reported quantitatively with respect to the effect of palliative RT on longitudinal Hr-QoL. In case of PROMs measuring a similar construct it will be assessed whether pooling of data appears appropriate and reasons for this decision will be noted (as described in (10)). A meta-analysis will be conducted by calculating summary statistics for individual studies and subsequently by calculating a combined intervention effect estimate if possible (as described in (11)). If this kind of meta-analysis should not be possible different ways of statistical analysis (e.g. summarising effect estimates) and visual display will be assessed for feasibility (as described in (12)). In case of sparse data at least a qualitative and narrative overview of PROM results will be provided separately for overall scores of multi-item questionnaires and for specific symptoms. The effect on Hr-QoL in dependence of a specific RT regime will be evaluated in case of i.) at least three or more studies using comparable dosing and fractionation and ii.) assessing a comparable PROM (e.g. comparable generic questionnaires for an overall score or same domain for single-item PROMs). However, in case of sparse data for this comparison, a narrative and qualitative assessment will be attempted. Any necessary deviation from the protocol plan will be explained.

### 2.7 Meta-biases

To reduce the risk of non-reporting bias up-front, the search strategy is designed as mentioned above. The risk of selective reporting of results within the studies retrieved will be assessed for the synthesis of studies reporting on overall scores of multi-item Hr-QoL PROMs. To do so, missing results in these studies will be screened and commented on. Finally, overall interpretation of meta-bias risk will be given.

### 2.8 Confidence in cumulative evidence

The strength of the body of evidence will be assessed according to GRADE-criteria.

## 3. Abbreviations

HNC, head and neck cancer; Hr-QoL, health-related quality of life; OS. overall survival; PROs, patient-reported outcomes; PROMs, patient-reported outcome measures; RT, radiotherapy

## 4. Appendix

1 - Search strategy

## 5. References

1. Ledeboer QCP, van der Schroeff MP, Pruyn JFA, de Boer MF, Baatenburg de Jong RJ, van der Velden L-A. Survival of patients with palliative head and neck cancer. Head Neck. 2011 Jul;33(7):1021–6.

2. Vermorken JB, Mesia R, Rivera F, Remenar E, Kawecki A, Rottey S, et al. Platinum-based chemotherapy plus cetuximab in head and neck cancer. N Engl J Med. 2008 Sep 11;359(11):1116–27.

3. Rischin D, Harrington KJ, Greil R, Soulieres D, Tahara M, de Castro G, et al. Protocol-specified final analysis of the phase 3 KEYNOTE-048 trial of pembrolizumab (pembro) as first-line therapy for recurrent/metastatic head and neck squamous cell carcinoma (R/M HNSCC). J Clin Oncol. 2019 May 20;37(15_suppl):6000–6000.

4. LeBlanc TW, Abernethy AP. Patient-reported outcomes in cancer care - hearing the patient voice at greater volume. Nat Rev Clin Oncol. 2017 Dec;14(12):763–72.

5. Shahid Iqbal M, Kelly C, Kovarik J, Goranov B, Shaikh G, Morgan D, et al. Palliative radiotherapy for locally advanced non-metastatic head and neck cancer: A systematic review. Radiother Oncol J Eur Soc Ther Radiol Oncol. 2018 Mar;126(3):558–67.

6. Chera BS, Eisbruch A, Murphy BA, Ridge JA, Gavin P, Reeve BB, et al. Recommended patient-reported core set of symptoms to measure in head and neck cancer treatment trials. J Natl Cancer Inst. 2014 Jul;106(7).

7. Rogers SN, Barber B. Using PROMs to guide patients and practitioners through the head and neck cancer journey. Patient Relat Outcome Meas. 2017;8:133–42.

8. Sterne JAC, Savović J, Page MJ, Elbers RG, Blencowe NS, Boutron I, et al. RoB 2: a revised tool for assessing risk of bias in randomised trials. BMJ. 2019 28;366:l4898.

9. Sterne JA, Hernán MA, Reeves BC, Savović J, Berkman ND, Viswanathan M, et al. ROBINS-I: a tool for assessing risk of bias in non-randomised studies of interventions. BMJ. 2016 Oct 12;355:i4919.

10. Johnston BC, Patrick DL, Devji T, Maxwell LJ, Bingham III CO, Beaton D, Boers M, Briel M, Busse JW, Carrasco-Labra A, Christensen R, da Costa BR, El Dib R, Lyddiatt, A, Ostelo RW, Shea B, Singh J, Terwee CB, Williamson PR, Gagnier JJ, Tugwell P, Guyatt GH. Chapter 18: Patient-reported outcomes. In: Higgins JPT, Thomas J, Chandler J, Cumpston M, Li T, Page MJ, Welch VA (editors). Cochrane Handbook for Systematic Reviews of Interventions version 6.0 (updated July 2019). In Cochrane; 2019.

11. Deeks JJ, Higgins JPT, Altman DG. Chapter 10: Analysing data and undertaking meta-analyses. In: Higgins JPT, Thomas J, Chandler J, Cumpston M, Li T, Page MJ, Welch VA (editors). Cochrane Handbook for Systematic Reviews of Interventions version 6.0. In Cochrane; 2019.

12. McKenzie JE, Brennan SE. Chapter 12: Synthesizing and presenting findings using other methods. In: Higgins JPT, Thomas J, Chandler J, Cumpston M, Li T, Page MJ, Welch VA (editors). Cochrane Handbook for Systematic Reviews of Interventions version 6.0. In Cochrane; 2019.
