# Supplementary material for: Patient-Reported Outcomes Assessing the Impact of Palliative Radiotherapy on Quality of Life and Symptom Burden in Head and Neck Cancer Patients: A Systematic Review
Source: Front Oncol. 2021 Jun 4;11:683042. doi: 10.3389/fonc.2021.683042 (PMC8213366; doi:10.3389/fonc.2021.683042)
Supplement: Supplementary file 2 [file DataSheet_2.docx]

**Protocol amendments**

**Patient-reported outcomes for health-related quality of life in head and neck cancer patients treated with palliative radiotherapy:
protocol of a systematic review and planned meta-analysis**

Alexander Fabian^1#^, Justus Domschikowski^1^, Markus Hoffmann^2^, Oliver Weiner^3^, Claudia Schmalz^1^, Jürgen Dunst^1^ and David Krug^1^

^1^ Department of Radiation Oncology, University Hospital Schleswig-Holstein Campus Kiel, Germany

^2^ Department of Otorhinolaryngology, Head and Neck Surgery, University Hospital Schleswig-Holstein Campus Kiel, Germany

^3^ University Library Kiel, Christian-Albrechts-University Kiel, Germany

# Correspondence address: Alexander Fabian MD, Department of Radiation Oncology, University Hospital Schleswig-Holstein (UKSH) Campus Kiel, Arnold-Heller-Str. 3, 24105 Kiel, Germany, Tel.: +4943150026557
Fax: +4943150026564, alexander.fabian@uksh.de

## Registration

The protocol was submitted in the “PROSPERO” registry on 22 January 2020 and published on 28 April 2020 (PROSPERO-ID: CRD42020166434). Amendments were submitted on 07 May 2020 and 11 November 2020 and await publication. Due to the high workload “PROSPERO” is facing during the SARS-CoV2 pandemic, documentation of final amendments to the protocol is restricted on the website. We therefore provide an updated version of relevant amendments to the protocol by this document following Cochrane recommendations.

## Amendments per protocol headings

2.1 Eligibility
i.) Head and neck cancer refers to squamous cell cancer of the head and neck (HNSCC).
ii.) “assessment of quality of life” was deleted as inclusion criterion. Reason: As initially planned, PRO on specific symptoms should also be evaluated. By deleting the aforementioned criterion the study population and search strategy did not change but study inclusion is more coherent and extensive as conceived initially.

2.2 Information sources
“EMBASE” was searched as planned initially and as reported in the manuscript. An interim modification not to cover EMBASE as stated in one of the updates on PROSPERO is no longer valid.

2.3 Study records
Studies reporting a “palliative effect” of palliative radiotherapy for squamous cell head and neck cancer were implicitly included into full text screening. This included retrospective as well as prospective studies.

2.4 Outcomes
Additional analysis:
Clinical studies of palliative radiotherapy for HNSCC reporting a "palliative effect" were counted at full text screening stage. These studies were analyzed for their use of patient reported-outcomes in order to provide a ratio of studies that state a "palliative effect" and do or do not use PRO. In case of PRO, the quality of patient-reported outcomes was assessed (validity, compliance rate, baseline measurement before RT, time of assessment reported).
A judgement on “palliative benefit” had to be stated in the abstract or discussion of studies included to full text screening. The semantic term of “palliative benefit” was assessed concisely. Terms like “effective palliation”, “significant palliative effect”, or “meaningful palliation” were counted, while studies solely stating terms like “improvement of symptoms” were not counted.

This additional analysis was decided post-hoc in order to gain information if statements on "palliative effects" are linked to their evaluation by PRO in this setting. It did neither require a modification to the search strategy nor to eligibility criteria for the primary research objective.

2.6 Data synthesis

A meta-analysis was deemed infeasible due to the paucity of studies and data. Instead, we conducted a narrative synthesis as suggested by Popay and colleagues (DOI: 10.13140/2.1.1018.4643). This method will be explained in detail in the manuscript.

### 
