# Supplementary material for: Patient-Reported Outcomes Assessing the Impact of Palliative Radiotherapy on Quality of Life and Symptom Burden in Head and Neck Cancer Patients: A Systematic Review
Source: Front Oncol. 2021 Jun 4;11:683042. doi: 10.3389/fonc.2021.683042 (PMC8213366; doi:10.3389/fonc.2021.683042)
Supplement: Supplementary file 4 [file Table_2.docx]

**Supplementary Table 2. Excluded publications based on full text screening.** The leading reason for exclusion is indicated. In some references, multiple reasons for exclusion may be present. Abbreviations: HNSCC: head and neck squamous cell carcinoma; PRO: patient-reported outcome; QoL: quality of life; RT: radiotherapy.

| **Reference** | **Reason for exclusion** |
| --- | --- |
| Agarwal JP, Nemade B, Murthy V, et al. Hypofractionated, palliative radiotherapy for advanced head and neck cancer. *Radiotherapy and Oncology* 2008; 89: 51–6 | No evidence of validity of PRO |
| Al-mamgani A, Tans L, Van rooij PH, et al. Hypofractionated radiotherapy denoted as the ‘Christie scheme’: an effective means of palliating patients with head and neck cancers not suitable for curative treatment. *Acta oncologica* 2009; 48: 562–70. | Not prospective (concerning QoL) |
| Al-Mamgani A, Kessels R, Verhoef CG, et al. Randomized controlled trial to identify the optimal radiotherapy scheme for palliative treatment of incurable head and neck squamous cell carcinoma. *Radiotherapy and Oncology* 2020; 149: 181–188 | No compliance data of PRO |
| Astrup GL, Rustøen T, Hofsø K, et al. Symptom burden and patient characteristics: Association with quality of life in patients with head and neck cancer undergoing radiotherapy. *Head and Neck* 2017; 39: 2114–2126. | No palliative RT |
| Barrett WL, Gleich L, Wilson K, et al. Organ preservation with interstitial radiation for base of tongue cancer. *American journal of clinical oncology* 2002; 25: 485–8. | No use of PRO |
| Basree MM, Mitchell DL, Dibs K, et al. Initial Experience With Palliative “QUAD-SHOT” Radiotherapy With Concurrent And Adjuvant PD-1 Inhibitor For Recurrent And/Or Metastatic Head And Neck Cancer. *International Journal of Radiation Oncology Biology Physics* 2020; 108: e826. | Not prospective |
| Carrascosa LA, Yashar CM, Paris KJ, et al. Palliation of pelvic and head and neck cancer with paclitaxel and a novel radiotherapy regimen. *Journal of Palliative Medicine* 2007; 10: 877–881. | No evidence of validity of PRO |
| Chen AM, Vaughan A, Narayan S, et al. Palliative radiation therapy for head and neck cancer: toward an optimal fractionation scheme. *Head & neck* 2008; 30: 1586–91. | Not prospective |
| Clarke S, Wilcox SW, Hill JD. Cyclical hypofractionated IMRT for effective palliation of head and neck cancers: Can modern techniques offer improved outcomes? *International Journal of Radiation Oncology Biology Physics* 2014; 90: S565. | Not prospective |
| Choudhary A GA. Conventional fractionation versus quad shot in advanced head-and-neck cancers: a randomized controlled trial. *Indian journal of palliative care* 2019; 25: 537–534. | No evidence of validity of PRO |
| Das S, Thomas S, Pal SK, et al. Hypofractionated Palliative Radiotherapy in Locally Advanced Inoperable Head and Neck Cancer: CMC Vellore Experience. *Indian Journal of Palliative Care* 2013; 19: 93–98. | No compliance data of PRO |
| Elting LS, Keefe DM, Sonis ST, et al. Patient-reported measurements of oral mucositis in head and neck cancer patients treated with radiotherapy with or without chemotherapy: demonstration of increased frequency, severity, resistance to palliation, and impact on quality of life. *Cancer* 2008; 113: 2704–13. | No palliative RT |
| Erkal HS, Mendenhall WM, Amdur RJ, et al. Squamous cell carcinomas metastatic to cervical lymph nodes from an unknown head and neck mucosal site treated with radiation therapy with palliative intent. *Radiotherapy and oncology : journal of the European Society for Therapeutic Radiology and Oncology* 2001; 59: 319–21. | Not prospective |
| Farina E, Capuccini J, Macchia G, et al. Phase I-II Study of Short-course Accelerated Radiotherapy (SHARON) for Palliation in Head and Neck Cancer. *Anticancer Research* 2018; 38: 2409–2414. | Not HNSCC (less than 50%) |
| Farina E, Capuccini J, Macchia G, et al. Short course accelerated radiation therapy (SHARON) in palliative treatment of advanced solid cancer in older patients: A pooled analysis. *Journal of Geriatric Oncology* 2018; 9: 359–361. | Duplicate |
| Fang FM, Tsai WL, Chien CY, et al. Changing quality of life in patients with advanced head and neck cancer after primary radiotherapy or chemoradiation. *Oncology* 2005; 68: 405–413. | No palliative RT |
| Ferro M, Macchia G, Cilla S, et al. Short-course accelerated palliative EBRT for advanced head and neck cancer in elderly patients. *Radiotherapy and Oncology* 2019; 133: S884–S885. | No compliance data of PRO |
| Gamez ME, Agarwal M, Hu KS, et al. Hypofractionated palliative radiotherapy with concurrent radiosensitizing chemotherapy for advanced head and neck cancer using the ‘QUAD-SHOT regimen’. *Anticancer Research* 2017; 37: 685–692. | Not prospective |
| Gamez ME, Hu K, Agarwal M, et al. Hypofractionated palliative radiation therapy with concurrent chemotherapy for advanced head-and-neck cancer: the quad-shot regimen management of recurrent head-and-neck squamous cell carcinoma. *International Journal of Radiation Oncology Biology Physics* 2014; 88: 508. | Duplicate |
| Gandia D, Wibault P, Guillot T, et al. Simultaneous chemoradiotherapy as salvage treatment in locoregional recurrences of squamous head and neck cancer. *Head & neck* 1993; 15: 8–15. | No use of PRO |
| Ghoshal S, Kuttikat PG, Kumar N, et al. Is Quad Shot regimen as effective as two week radiotherapy for palliation of advanced head and neck carcinoma? *Oral Oncology* 2011; S55. | No evidence of validity of PRO |
| Ghoshal S, Chakraborty S, Moudgil N, et al. Quad shot: a short but effective schedule for palliative radiation for head and neck carcinoma. *Indian Journal of Palliative Care* 2009; 15: 137–140. | No evidence of validity of PRO |
| Hammerlid E, Silander E, Hornestam L, et al. Health-related quality of life three years after diagnosis of head and neck cancer--a longitudinal study. *Head & neck* 2001; 23: 113–25. | No palliative RT |
| Haraf DJ, Vokes EE, Panje WR, et al. Survival and analysis of failure following hydroxyurea, 5-fluorouracil and concomitant radiation therapy in poor prognosis head and neck cancer. *American journal of clinical oncology* 1991; 14: 419–26. | No use of PRO |
| Jakhar SL, Purohit R, Solanki A, et al. Accelerated hypofractionation (OCTA SHOT): Palliative radiation schedule in advanced head and neck carcinoma. *Journal of Cancer Research and Therapeutics* 2017; 13: 943–946. | No use of PRO |
| Jensen K, Schmidt HH, Jensen AB, et al. Effect of short course palliative radiotherapy (RT) for head and neck cancer: A prospective single institution quality of life (QoL) trial. *European Journal of Cancer* 2013; 49: S759. | No compliance data of PRO |
| Kancherla KN, Oksuz DC, Prestwich RJ, et al. The role of split-course hypofractionated palliative radiotherapy in head and neck cancer. *Clinical Oncology* 2011; 23: 141–8. | Not prospective |
| Kanti Pal S, Das S, Thomas S, et al. Feasibility of a novel palliative radiotherapy schedule in advanced, inoperable head and neck cancer: A quality of life study. *Journal of Cancer Research and Therapeutics* 2012; 8: S175. | Duplicate |
| Kaushal V, Soni A, Dhull A K. Comparative evaluation of two schedules of palliative radiotherapy in locally advanced head and neck carcinoma. *Journal of cancer research and therapeutics* 2012; 8: 166. | No use of PRO |
| Kaushal V, Soni A, Dhull A K. Comparative evaluation of two schedules of palliative radiotherapy in locally advanced head and neck carcinoma. *Journal of cancer research and therapeutics* 2012; 8: 166. | Duplicate |
| Khan S, Kamble K M, Diwan A K, et al. Palliative RT in locally very advanced head and neck cancers if chemoresistance is a harbinger of radioresistance, can it be circumvented by a hybrid fractionation schedule? *Journal of cancer research and therapeutics 12(6):S53-, 2016*; 12: 53. | No use of PRO |
| Khan L, Tjong M, Raziee H, et al. Role of stereotactic body radiotherapy for symptom control in head and neck cancer patients. *Supportive care in cancer* 2015; 23: 1099–103. | Not prospective |
| Kolotas C, Tselis N, Sommerlad M, et al. Reirradiation for recurrent neck metastases of head-and-neck tumors using CT-guided interstitial 192Ir HDR brachytherapy. *Strahlentherapie und Onkologie* 2007; 183: 69–75. | No use of PRO |
| Kumar A, Sharma A, Mohanti B K, et al. A phase II randomized study to compare short course palliative radiotherapy with short-course concurrent palliative chemotherapy plus radiotherapy in advanced and unresectable head and neck cancer. *European journal of cancer Conference: European Cancer Congress 2013, ECC 2013* 2013; 751. | Duplicate (3x) |
| Kumar A, Sharma A, Mohanti BK, et al. A phase 2 randomized study to compare short course palliative radiotherapy with short course concurrent palliative chemotherapy plus radiotherapy in advanced and unresectable head and neck cancer. *Radiotherapy and Oncology* 2015; 117: 145–151. | No use of PRO |
| Kumaravelu AS, Eswaran P. Palliative hypofractionated radiotherapy and chemotherapy in advanced head and neck squamous cell carcinoma (HNSCC). *Supportive Care in Cancer* 2012; 20: S62. | Time of assessment of PRO not reported |
| Lok BH, Jiang G, Gutiontov S, et al. Palliative head and neck radiotherapy with the RTOG 8502 regimen for incurable primary or metastatic cancers. *Oral Oncology* 2015; 51: 957–962. | Not prospective |
| Lok BH, Riaz N, Lanning RM, et al. Palliative head and neck radiation therapy with the quad shot regimen for incurable primary or metastatic cancers. *International Journal of Radiation Oncology Biology Physics* 2014; 90: S700. | Duplicate |
| Manik VD. Split-course hypofractionated radiotherapy for palliation of advanced head and neck squamous cell carcinoma. *Journal of Cancer Research and Therapeutics* 2014; 10: S25. | No evidence of validity of PRO |
| Minatel E, Gigante M, Franchin G, et al. Combined radiotherapy and bleomycin in patients with inoperable head and neck cancer with unfavourable prognostic factors and severe symptoms. *Oral Oncology* 1998; 34: 119–122. | No use of PRO |
| Mohanti BK, Umapathy H, Bahadur S, et al. Short course palliative radiotherapy of 20 Gy in 5 fractions for advanced and incurable head and neck cancer: AIIMS study. *Radiotherapy and Oncology* 2004; 71: 275–280. | No evidence of validity of PRO |
| Mudgal A, Arya AK, Yadav I, et al. Role of hypofractionated palliative radiotherapy in patients with stage four head-and-neck squamous cell carcinoma. *Journal of Cancer Research and Therapeutics* 2019; 15: 528–532. | No evidence of validity of PRO |
| Murthy V, Kumar D, Budrukkar A, et al. Twice-weekly palliative radiotherapy in locally very advanced, incurable head and neck cancers. *Oral Oncology* 2013; 49: S138. | Duplicate |
| Murthy V, Kumar D, Budrukkar A, et al. Twice-weekly palliative radiotherapy for locally very advanced head and neck cancers. *Indian Journal of Cancer* 2016; 53: 138–141. | No evidence of validity of PRO |
| Nguyen N, Hodson I, Doerwald-Munoz L, et al. Retrospective study of hypofractionated palliative radiation therapy for advanced head-and-neck cancers 0-7-21. *International Journal of Radiation Oncology Biology Physics* 2012; 84: S521. | Duplicate |
| Nguyen NT, Doerwald-Munoz L, Zhang H, et al. 0-7-21 hypofractionated palliative radiotherapy: an effective treatment for advanced head and neck cancers. *The British journal of radiology* 2015; 88: 20140646. | Not prospective |
| Nguyen NTA, Doerwald-Munoz L, Wright J, et al. Phase 2 study of “0-7-21” hypofractionated palliative radiation therapy for advanced head and neck squamous cell carcinoma. *International Journal of Radiation Oncology Biology Physics* 2015; 93: S128. | No compliance data of PRO |
| Ninu MB, Miccinesi G, Bulli F, et al. Psychological distress and health-related quality of life among head and neck cancer patients during the first year after treatment. *Tumori* 2016; 102: 96–102. | No palliative RT |
| Paliwal R, Patidar AK, Walke R, et al. Palliative hypofractionated radiotherapy in locally advanced head and neck cancer with fixed neck nodes. *Iranian Journal of Cancer Prevention* 2012; 5: 178–182. | No evidence of validity of PRO |
| Pandey K, Revannasiddaiah S, Pant N K, et al. Palliative radiotherapy in locally advanced head and neck cancer after failure of induction chemotherapy: comparison of two fractionation schemes. *Indian Journal of Palliative Care* 2013; 19: 139–145. | No evidence of validity of PRO |
| Paris KJ, Spanos WJ Jr, Lindberg RD, et al. Phase I-II study of multiple daily fractions for palliation of advanced head and neck malignancies. *International journal of radiation oncology, biology, physics* 1993; 25: 657–60. | No evidence of validity of PRO |
| Pearson RA, Bannister-Young RH, Ivison D, et al. Split-course hypofractionated palliative radiotherapy for patients with head and neck squamous cell carcinoma - a worthwhile treatment schedule in the UK? *Clinical oncology* 2010; 22: 890–1. | Not prospective |
| Pilepich MV, Munzenrider JE, Rene JB. Unorthodox fractionation in the treatment of head and neck tumors. *International journal of radiation oncology, biology, physics* 1979; 5: 249–52. | No use of PRO |
| Puthawala A, Nisar Syed AM, Gamie S, et al. Interstitial low-dose-rate brachytherapy as a salvage treatment for recurrent head-and-neck cancers: long-term results. *International journal of radiation oncology, biology, physics* 2001; 51: 354–62. | No palliative RT |
| Rahn AN, Schilcher RB, Adamietz IA, et al. [Palliative radiochemotherapy with Bendamustin for advanced recurrent head and neck tumors]. *Strahlentherapie und Onkologie* 2001; 177: 189–94. | Language other than English |
| Rahn AN, Schilcher RB, Adamietz IA, et al. Palliative radiochemotherapy with bendamustin in locally advanced recurrent tumors of the head and neck. *Strahlentherapie und Onkologie* 2001; 177: 189–194. | Duplicate |
| Rich SE, Mendenhall WM. Rapid Radiation Therapy for Advanced Cancer of the Head and Neck #336. *Journal of Palliative Medicine* 2017; 20: 1034–1035. | No clinical study (review) |
| Saroja KR, Hendrickson FR, Cohen L, et al. Re-irradiation of locally recurrent tumors with fast neutrons. *International journal of radiation oncology, biology, physics* 1988; 15: 115–21. | Not prospective |
| Schleicher UM, Andreopoulos D, Ammon J. Palliative radiotherapy in recurrent head-and-neck tumors by a percutaneous superfractionated treatment schedule. *International journal of radiation oncology, biology, physics* 2001; 50: 65–8. | No evidence of validity of PRO |
| Schwab W, Ries G, Handschuh K. [Initial experiences with interstitial brachycurietherapy (low dose rate I-125 seeds in carrier/vicryl and high dose rate Ir-192 afterloading system) in the palliative treatment of head and neck tumors]. *HNO* 1986; 34: 327–33. | Language other than English |
| Singh C, Gupta S. Palliative radiotherapy in locally advanced head and neck cancer: A quality of life study. *Supportive Care in Cancer* 2014; 22: S187. | No compliance data of PRO |
| Soni A, Kaushal V, Verma M, et al. Comparative evaluation of three palliative radiotherapy schedules in locally advanced head and neck cancer. *World journal of oncology* 2017; 8: 7–14. | No evidence of validity of PRO |
| Spaeth J, Andreopoulos D, Unger T, et al. Intra-operative radiotherapy - 5 years of experience in the palliative treatment of recurrent and advanced head and neck cancers. *Oncology* 1997; 54: 208–213. | No evidence of validity of PRO |
| Spaeth J, Andreopoulos D, Unger T. [Palliative intraoperative irradiation of recurrent neck lymph node metastases in the head and neck area]. *Laryngo- rhino- otologie* 1997; 76: 36–41. | Language other than English |
| Spaeth J, Andreopoulos D, Unger T. Palliative intraoperative radiation therapy (IORT) of recurrent metastases in lymph nodes in the head and neck region. *Laryngo- Rhino- Otologie* 1997; 76: 36–41. | Duplicate |
| Suwinski R, Pilecki B, Skladowski K, et al. Sequential combination of radio-chemotherapy (5-fluorouracil, cis-platinum and irradiation) in the management of locally advanced head and neck cancers. *Neoplasma* 1996; 43: 37–41. | No evidence of validity of PRO |
| Tercilla OF, Schmidt-Ullrich R, Wazer DE. Reirradiation of head and neck neoplasms using twice-a-day scheduling. *Strahlentherapie und Onkologie* 1993; 169: 285–90. | No use of PRO |
| Tselis N, Karagiannis E, Kolotas C, et al. Image-guided interstitial high-dose-rate brachytherapy in the treatment of inoperable recurrent head and neck malignancies: An effective option of reirradiation. *Head & neck* 2017; 39: E61-e68. | Case report |
| Tselis N, Ratka M, Vogt HG, et al. Hypofractionated accelerated CT-guided interstitial (1)(9)(2)Ir-HDR-Brachytherapy as re-irradiation in inoperable recurrent cervical lymphadenopathy from head and neck cancer. *Radiotherapy and Oncology* 2011; 98: 57–62. | Not prospective |
| van Beek KM, Kaanders JH, Janssens GO, et al. Effectiveness and toxicity of hypofractionated high-dose intensity-modulated radiotherapy versus 2- and 3-dimensional radiotherapy in incurable head and neck cancer. *Head & neck* 2016; 38 Suppl 1: E1264-70. | Not prospective |
| van Beek KM, Kaanders JHAM, Janssens GORJ, et al. Hypofractionated high-dose IMRT: Effective and less toxic than 2D-RT in incurable head and neck cancer. *Radiotherapy and Oncology* 2015; 115: S383–S384. | Not prospective |
| Vijayakumar S. SG. Palliative radiotherapy in advanced head and neck malignancy with high dose schedule. *Indian Journal of Radiology & Imaging* 1991; 1: 371–3. | Full text not retrievable |
| Vikram B, Hilaris BS, Anderson L, et al. Permanent Iodine-125 implants in head and neck cancer. *Cancer* 1983; 51: 1310–4. | Not prospective |
| Voynov G, Heron DE, Burton S, et al. Frameless stereotactic radiosurgery for recurrent head and neck carcinoma. *Technology in cancer research & treatment* 2006; 5: 529–35. | Not prospective |
| Weissberg JB, Pillsbury H, Sasaki CT, et al. High fractional dose irradiation of advanced head and neck cancer. Implications for combined radiotherapy and surgery. *Archives of otolaryngology* 1983; 109: 98–102. | No use of PRO |
| Weissberg JB, Son YH, Percarpio B, et al. Randomized trial of conventional versus high fractional dose radiation therapy in the treatment of advanced head and neck cancer. *International journal of radiation oncology, biology, physics* 1982; 8: 179–85. | Duplicate (2x) |
| Zamboglou N, Wurm R, Pape H, et al. Simultaneous radiotherapy and intratumoral instillation of mitoxantrone in locoregional recurrence of head and neck carcinomsa. *Regional Cancer Treatment* 1991; 4: 79–84. | No evidence of validity of PRO |
