# Supplementary material for: Patient-Reported Outcomes Assessing the Impact of Palliative Radiotherapy on Quality of Life and Symptom Burden in Head and Neck Cancer Patients: A Systematic Review
Source: Front Oncol. 2021 Jun 4;11:683042. doi: 10.3389/fonc.2021.683042 (PMC8213366; doi:10.3389/fonc.2021.683042)
Supplement: Supplementary file 5 [file Table_3.docx]

**Supplementary Table 3. Excluded registered trials based on full text screening.** The year of the start of a specific trial is indicated. HNSCC: head and neck squamous cell carcinoma; PRO: patient-reported outcome; QoL: quality of life; RT: radiotherapy.

| **Reference** | **Reason for exclusion** |
| --- | --- |
| CTRI/2012/06/002717. We are comparing two arm of treatment ,compare short course palliative Radiotherapy with short course concurrent palliative Chemotherapy plus radiotherapy in advanced and unresectable head and neck cancer. *Clinical Trials Registry India 2012* | Terminated and published. Publication was included in screening process. |
| NCT02421458. Dutch Randomized Multicenter Trial COmparing twO PalliativE RAdiaTION Schemes for Incurable Head and Neck Cancer (COOPERATION). *ClinicalTrials.gov* 2015. | Terminated and published. Publication was included in screening process. |
| NCT02460471. Palliative Radiotherapy Protocol in Head and Neck Cancer. *ClinicalTrials.gov* 2011. | Terminated and published. Publication was included in screening process. |
| NCT03196700. Short Course Radiation Therapy in Palliative Treatment. *ClinicalTrials.gov* 2009. | Terminated and published. Publication was included in screening process. |
| NCT03218475. MRG FU With Radiotherapy for Palliation of H&N Cancer. *ClinicalTrials.gov* 2016. | No use of PRO |
| NCT03317327. Reirradiation and Programmed Cell Death Protein 1 (PD-1) Blockade On Recurrent Squamous Cell Head and Neck Tumors. *ClinicalTrials.gov* 2017. | No use of PRO |
| NCT03637335. Comparing Palliative Radiotherapy With or Without Carboplatin. *ClinicalTrials.gov* 2015. | Registered study, early termination |
| NCT03804073. Short Course Radiation Therapy in Palliative Treatment of Head and Neck Cancer. *ClinicalTrials.gov* 2017. | Ongoing study |
| Optimum radiation dose for palliation in head and neck Squamous cell carcinoma A[cents]?? A phase 3 randomized controlled trial (OpRaH). *http://www.who.int/trialsearch/Trial2.aspx?TrialID=CTRI*; 06, 2020 | Fulltext not retrievable |
